# Causal association between skin cancer and immune cells: mendelian randomization (MR) study

**DOI:** 10.1186/s12885-024-12603-0

**Published:** 2024-07-17

**Authors:** Wei Yin, Ruilei Li, Zhaoqi Zhang, Ying Wang, Xinghua Tang, Lin Zhu, Hong Yao, Ke Li

**Affiliations:** Cancer Biotherapy Center, Key Laboratory of Melanoma Research, The Third Affiliated Hospital of Kunming Medical University (Tumor Hospital of Yunnan Province), Kunming, Yunnan Province 650000 China

**Keywords:** Mendelian randomization, Immune cells, Skin Cancer, MM, BCC, AK, SCC

## Abstract

**Background:**

Numerous meta-analyses and clinical studies have shown that subtypes of immune cells are associated with the development of skin cancer, but it is not clear whether this association is causal or biased. Mendelian randomization (MR) analysis reduces the effect of confounding factors and improves the accuracy of the results when compared to traditional studies. Thus, in order to examine the causal relationship between various immune cell and skin cancer, this study employs two-sample MR.

**Methods:**

This study assesses the causal association between 731 immune cell characteristics and skin cancer using a two-sample Mendel randomization (MR) methodology. Multiple MR methods were used to bias and to derive reliable estimates of causality between instrumental variables and outcomes. Comprehensive sensitivity analyses were used to validate the stability, heterogeneity and horizontal multiplicity of the results.

**Results:**

We discovered that potential causal relationships between different types of immune cells and skin cancer disease. Specifically, one type of immune cell as potentially causal to malignant melanoma of skin (MM), eight different types of immune cells as potentially causal to basal cell carcinoma (BCC), four different types of immune cells as potentially causal to actinic keratosis (AK), and no different types of immune cells were found to have a potential causal association with squamous cell carcinoma(SCC), with stability in all of the results.

**Conclusion:**

This study demonstrates the close connection between immune cells and skin cancer disease by genetic means, which enriches the current knowledge about the role of immune cells in skin cancer and also contributes to the design of therapeutic strategies from an immunological perspective.

**Supplementary Information:**

The online version contains supplementary material available at 10.1186/s12885-024-12603-0.

## Introduction

Skin cancer is a widespread type of cancer worldwide, primarily caused by mutations, abnormal differentiation, and irregular nuclear division of epidermal keratinocytes. According to the origin of tumor cells, it can be divided into three categories: melanoma (MM), basal cell carcinoma (BCC), and squamous cell carcinoma (SCC) [1]. Actinic keratosis (AK) is a precancerous lesion characterized by aberrant proliferation of keratinocytes, often considered a precursor to SCC [2]. BCC and SCC account for the majority of skin cancer cases, approximately 95% [1], while MM represents only 2% of skin cancer cases. However, MM has a high risk of early metastasis and a mortality rate as high as 80% [3]. According to World Health Organization statistics, in 2020, there were nearly 1.2 million new cases of BCC and SCC globally and 330,000 new cases of MM [4]. The exact causes of skin cancer remain unclear and may be associated with factors such as exposure to ultraviolet radiation, chronic irritation, immunity, and genetics. Currently, in addition to surgical excision, common treatments for skin cancer include targeted therapy and immunotherapy. However, tumor resistance often leads to treatment failure in a significant number of patients [5, 6]. Hence, there is an urgent need to explore novel treatment strategies and develop innovative therapeutic drugs.

Multiple relevant studies have indicated that the immune system plays a pivotal role in the occurrence, growth, and metastasis of malignancies [7]. The skin, as an integral component of the innate immune system, serves as a protective barrier against external threats. When this barrier is breached, innate immune cells and adaptive immune cells are promptly activated. They mount a swift and effective response, both non-antigen-specific and antigen-specific, aimed at eliminating the invading pathogens [8]. Furthermore, the immune system plays a crucial role in detecting and destroying tumor cells. The process of skin immune surveillance is primarily mediated by antigen-presenting cells (APCs). These APCs have the ability to enter the skin-draining lymph nodes, where they present antigens to T cells, ultimately initiating immune responses [9, 10]. Bacci et al. [11]. discovered that UV radiation diminishes the number of APCs that migrate to the skin-draining lymph nodes, thereby impairing APC function. APCs present antigens to cytotoxic T cells (CD8+) through HLA I. Subsequently, co-stimulatory molecules such as CD28 on T cells and B7.1 on APCs activate cytotoxic T cell responses [11, 12]. Furthermore, macrophages play a significant role in the occurrence and development of tumors. Low levels of interferon-γ and high levels of IL-4 promote macrophage differentiation into M2, facilitating tumor progression. Meanwhile, CD4 + T cells downregulate interferon-γ and IL-2, increase the production of IL-4 and IL-5, further promoting tumor development [13]. Besides protecting the body from pathogen invasion, the immune system also prevents tumor development and eliminates malignant cells. Exploring the function and mechanisms of the immune system in skin cancer can provide new insights for treatment methods, improving the treatment and prognosis of malignant skin tumor patients. However, up to date, research results on the relationship between immune cells and skin cancer have been inconsistent, possibly due to limited sample sizes, study design flaws, and confounding factors beyond the scope of existing research.

**Fig. 1:**
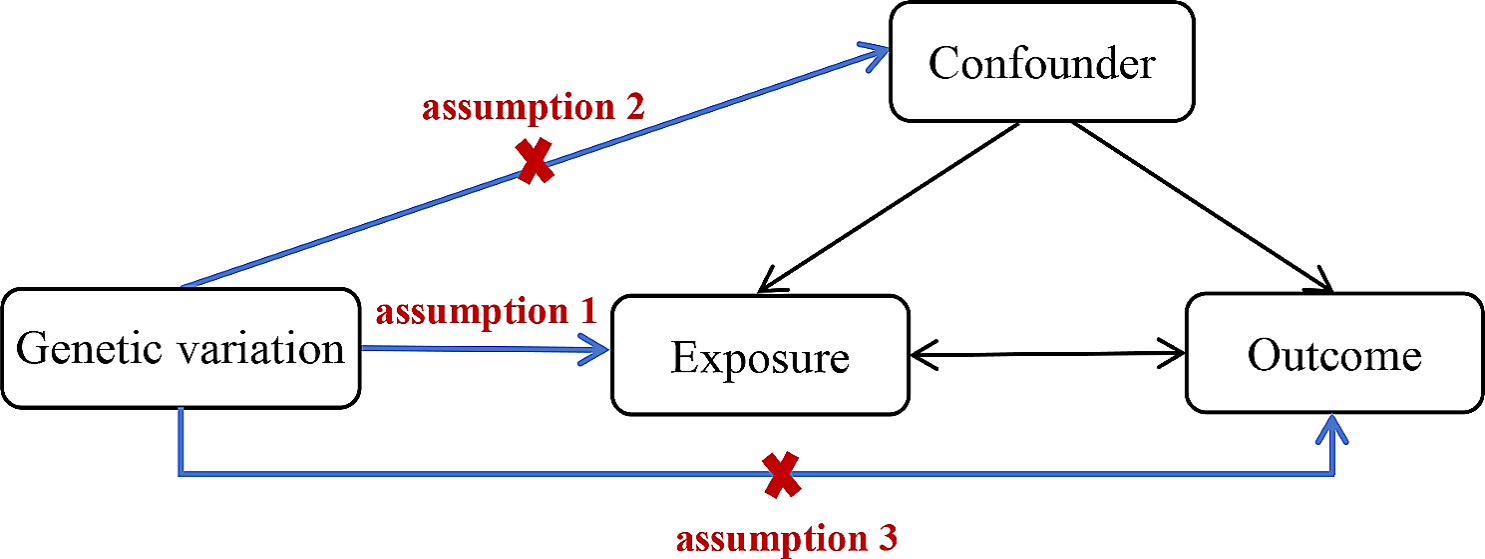
The three key assumptions of MR

Mendelian randomization (MR) is a statistical approach rooted in genetic variation, utilized to ascertain causal connections between exposures or risk factors and clinically relevant outcomes [14]. MR employs genetic variants intimately linked to the exposure level within the population as instrumental variables (IVs), simulating the conditions of random allocation in experiments. This method enables the identification of causal relationships untainted by confounding factors and guards against reverse causation [15]. This type of analysis relies on three core assumptions: (1) genetic variation is directly associated with the exposure; (2) genetic variation is not associated with confounding factors between the exposure and the outcome; (3) genetic variation does not affect the outcome through mechanisms other than the exposure [16] (Fig. 1).

**Fig. 2:**
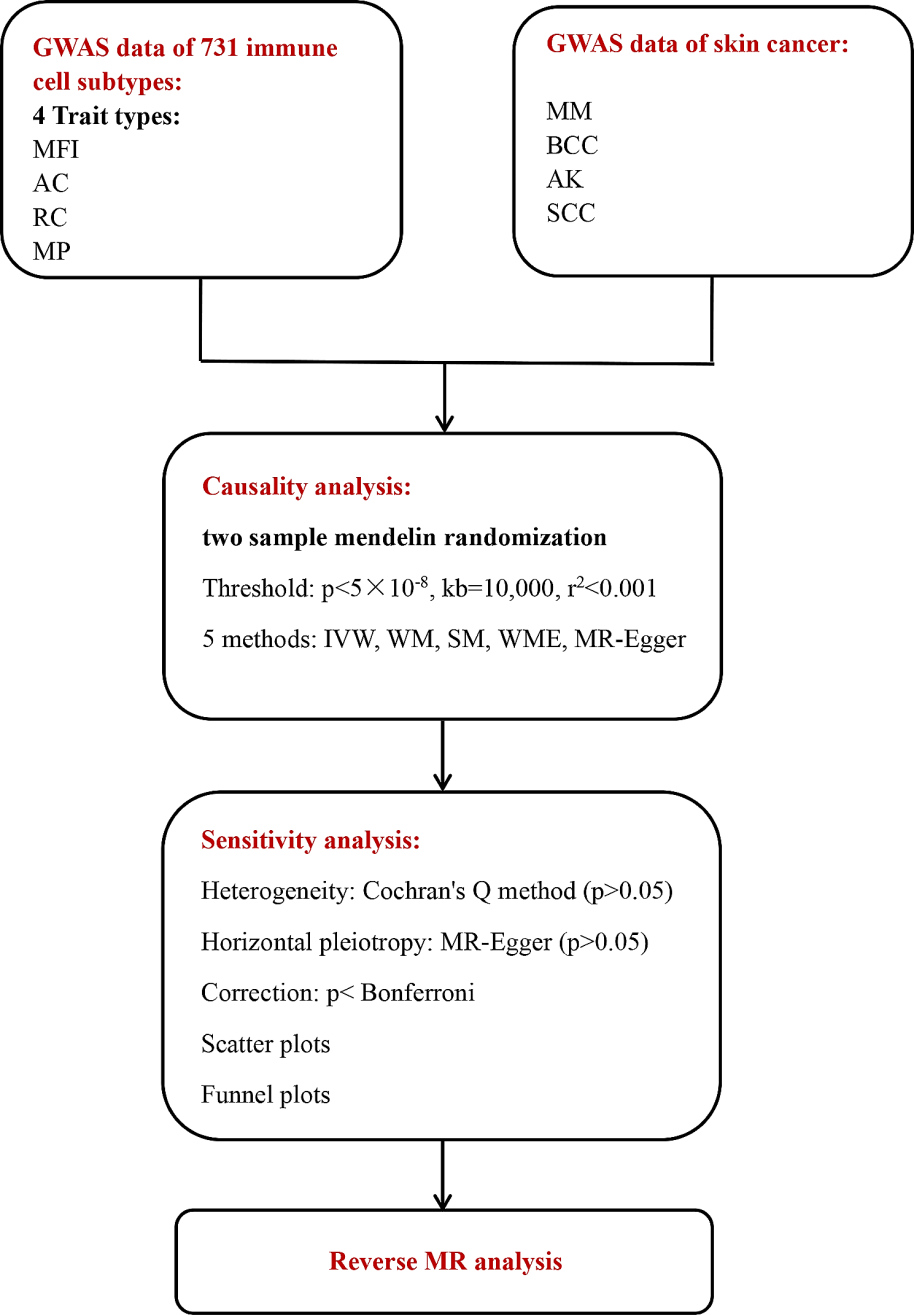
Workflow of our research

Therefore, this study utilized the MR method to analyze the causal relationship between different types of immune cell phenotypes and skin cancer in the European population. Two-sample MR analyses were conducted to examine potential causal relationships between different types of immune cells and the risk of skin cancers such as MM, SCC, AK, and BCC. This study aims to provide new strategies for the prevention and treatment of skin cancer diseases. The workflow is shown in Fig. 2.

## Materials and methods

### Research design

Using a two-sample Mendelian randomization (MR) approach, we conducted an analysis to assess the causal relationships between 731 immune cell subtypes and skin cancer. MR analysis typically utilizes genetic variation as instrumental variables to assess the causal impact of exposures on outcomes. As this study was based on publicly available data, no additional ethical approval or consent was required.

### Data sources for exposure and outcome

#### GWAS data sources for skin cancer

In this study, skin cancer diseases include MM, BCC, SCC, and AK. The data retrived from the GWAS website (https://gwas.mrcieu.ac.uk/). For MM, the genetic data are from Neale et al., who conducted a GWAS on 361,194 Europeans (Ncase = 2,534, Ncontrol = 358,660), identifying a total of 10,855,955 SNPs. The genetic data for BCC are from Adolphe et al. [17], who performed a GWAS on 392,971 Europeans (Ncase = 17,416, Ncontrol = 375,455), confirming 71 GWAS loci and 46 functional candidate BCC susceptibility genes. Among these genes, 26 with decreased expression and 20 with increased expression were associated with an increased risk of BCC, totaling 7,244,167 SNPs. The genetic data for SCC are from the Neale Lab, which conducted a GWAS on 337,159 Europeans (Ncase = 404, Ncontrol = 336,755), identifying a total of 10,894,596 SNPs. In the genetic data for AK, researchers performed a GWAS on 218,090 Europeans (Ncase = 4,817, Ncontrol = 213,273), identifying a total of 16,380,463 SNPs.

#### GWAS data sources for immune cells

In this research, we obtained publicly available GWAS data for immune cells from the GWAS catalog and conducted an extensive analysis of genetic variations in a population of 3,757 individuals from Sardinia, evaluating 731 immune cell types to identify genetic variations associated with immune cell characteristics. Through a large-scale analysis of genetic variations at 70 loci, including 53 newly discovered, and 459 cellular traits in these Sardinian individuals, we identified 122 significantly independent association signals. This identification provides insights into several molecules and mechanisms involved in cellular regulation. Flow cytometry analysis encompassed 118 absolute cell (AC) counts, 389 median fluorescence intensities (MFI) reflecting surface antigen levels, 32 morphological parameters (MP), and 192 relative cell (RC) counts. Specifically, MFI, AC, and RC features included B cells, CDCs, mature stage T cells, monocytes, myeloid cells, TBNK (T cells, B cells, natural killer cells), and Treg cells. Meanwhile, MP features included CDC and TBNK cells [18, 19].

### Instrument selection

To identify SNPs associated with the exposure factor and ensure the reliability and accuracy of conclusions regarding the relationship between immune cells and skin cancer risk, the following steps were taken to select the most optimal SNPs. Initially, acknowledging that only a limited number of SNPs in the immune cell group met the genome-wide significance threshold (*p* < 5 × 10^− 8^). Additionally, to ensure the independence of the selected instrumental variables (IVs) and minimize bias resulting from residual linkage disequilibrium of genetic variations, we employed the two-sample MR R package with a distance set at 10,000 kb and a linkage disequilibrium threshold of r^2^ < 0.001. On top of that, to mitigate the potential bias resulting from weak instrumental variables, the F-statistic was utilized to assess the statistical strength of the correlation between each SNP and the exposure. IVs with an F-statistic exceeding 10 were considered strong instruments, while those with an F-statistic less than 10 indicated a weak correlation between SNPs and the exposure. During the analysis, SNPs with palindromic structures were automatically excluded. The F-statistic calculation employed the formula *F = R*^*2*^*/(1-R*^*2*^*)∙(N-K-1)/K*, where *N* signifies the sample size of the GWAS, K represents the number of single nucleotide polymorphisms (SNPs), *R*^*2*^ reflects the proportion of variance explained by SNPs in the exposure database, $$\:{R}^{2}=2\times\:\left(1-MAF\right)\times\:MAF\times\:\raisebox{1ex}{$\beta\:$}\!\left/\:\!\raisebox{-1ex}{$SD$}\right.$$. MAF is the minor allele frequency (equivalent to the frequency of the effect allele), and β is the effect value of the allele [20].

### Statistical analysis

All statistical analyses were implemented by the package Two-Sample MR (version 0.5.6) and Radial MR (version 1.0) in R (version 4.2.1).

In this study, we applied five MR methods to validate the causal associations between genetic variations in immune cells and MM, BCC, SCC, and AK. The methods comprised inverse variance weighted analysis (IVW), weighted median (WM), simple median (SM), weighted median estimator (WME), and MR-Egger regression. In the analysis, the IVW method, based on genotype summary data, served as the primary approach [21]. The IVW method combined Wald estimates for each single nucleotide polymorphism through a meta-analysis, resulting in an overall estimate. The weighted regression slope of the effect of the result on the effect of the exposure, with the intercept constrained to zero, represented the overall estimate. This approach provided a comprehensive evaluation of the causal relationship between genetic variations in immune cells and MM, BCC, SCC, and AK.

In secondary sensitivity analysis, we employed Cochran’s Q method to assess the heterogeneity of the selected IVs [22]. A significant result (*p* < 0.05) would indicate significant heterogeneity in the analysis outcomes. To reduce the influence of horizontal pleiotropy, we utilized the MR-Egger regression test [23]. If a significant intercept term is observed (*p* < 0.05), it suggests the presence of horizontal pleiotropy. Additionally, we applied the Bonferroni method for correction, considering only results with *p*-values < Bonferroni value in the final analysis. The Bonferroni correction formula is 0.05 / (number of exposures included in the study * number of outcomes included in the study) [24].

Finally, in order to explore whether skin cancer diseases have any causal relationship with established important immune cells, we conducted a reverse MR analysis using SNPs related to skin cancer diseases as IVs (skin cancer diseases as exposure and established immune cells as outcomes).

## Results

After the selection of instrumental variables (IVs), potential causal relationships were identified between one type of immune cell and MM, eight different types of immune cells and BCC, four different types of immune cells and actinic keratosis AK, no different types of immune cells were found to have a potential causal association with SCC. All IVs had F-statistics greater than 10, indicating the absence of weak instrument bias. Following Bonferroni correction, all *p*-values were below the Bonferroni threshold.

### Causal relationship between immune cells and MM

**Fig. 3:**
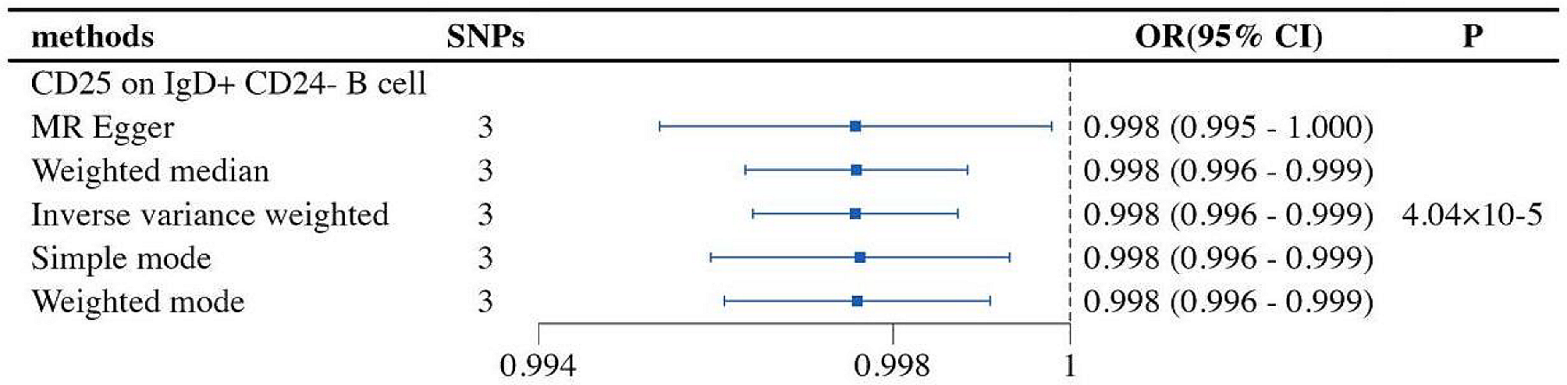
The forest plot shows the causal relationship between immune cell traits and MM

Our study revealed a potential causal relationship between one type of immune cell and melanoma (MM). Specifically, an increase in the abundance of CD25 on IgD + CD24- B cells was negatively correlated with the risk of MM (OR = 0.998, 95% CI = 0.996-1.000, *p* = 4.04E-05). In the reverse MR analysis of immune cells and MM, all MR results were greater than 0.05, indicating that MM had no impact on the included immune cells. The final results demonstrate a potential causal relationship between one type of immune cell and MM (Fig. 3).

### Causal relationship between immune cells and BCC

**Fig. 4:**
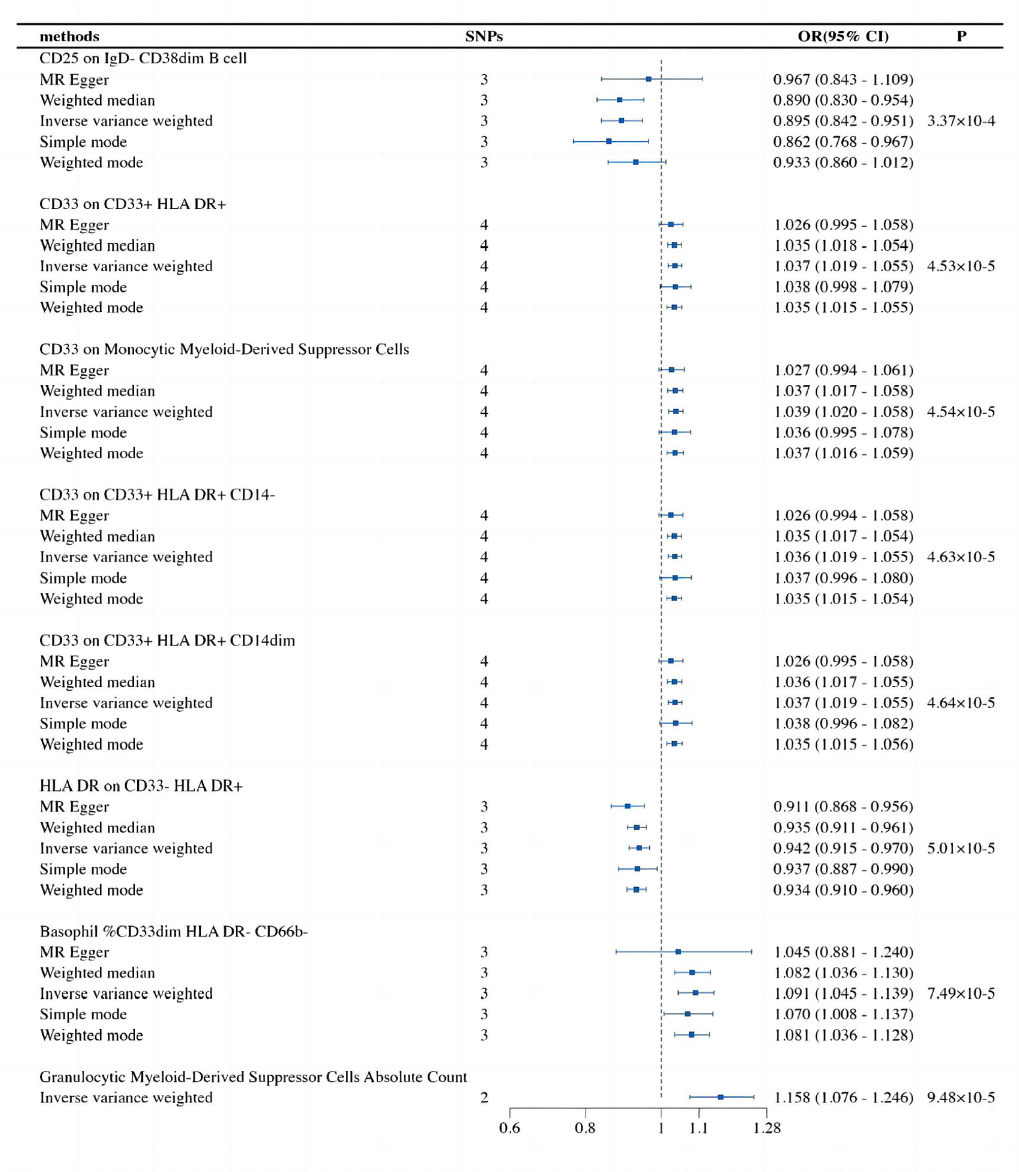
The forest plot shows the causal relationship between immune cell traits and BCC

In our study, we revealed that eight types of immune cells were potentially causally associated with BCC. Among them, an increase in the abundance of CD25 on IgD- CD38dim B cells and HLA DR on CD33- HLA DR + was negatively correlated with the risk of BCC, while the remaining immune cells showed a positive correlation. The IVW analysis results are as follows: CD25 on IgD- CD38dim B cell (OR = 0.895, 95% CI = 0.842–0.951, *p* = 3.37E-04), CD33 on CD33 + HLA DR+ (OR = 1.037, 95% CI = 1.019–1.055, *p* = 4.53E-05), CD33 on Monocytic Myeloid-Derived Suppressor Cells (OR = 1.039, 95% CI = 1.020–1.059, *p* = 4.54E-05), CD33 on CD33 + HLA DR + CD14- (OR = 1.036, 95% CI = 1.019–1.055, *p* = 4.63E-05), CD33 on CD33 + HLA DR + CD14dim (OR = 1.037, 95% CI = 1.019–1.055, *p* = 4.64E-05), HLA DR on CD33- HLA DR+ (OR = 0.942, 95% CI = 0.915–0.970, *p* = 5.01E-05), Basophil %CD33dim HLA DR- CD66b- (OR = 1.091, 95% CI = 1.045–1.140, *p* = 7.49E-05), Granulocytic Myeloid-Derived Suppressor Cells Absolute Count (OR = 1.158, 95% CI = 1.076–1.255, *p* = 9.48E-05). Finally, in the reverse MR analysis of all immune cells and BCC, all MR results were greater than 0.05, indicating that BCC had no effect on the included immune cells. The ultimate results demonstrate potential causal relationships between eight phenotypes of immune cells and BCC (Fig. 4).

### Causal relationship between immune cells and AK

**Fig. 5:**
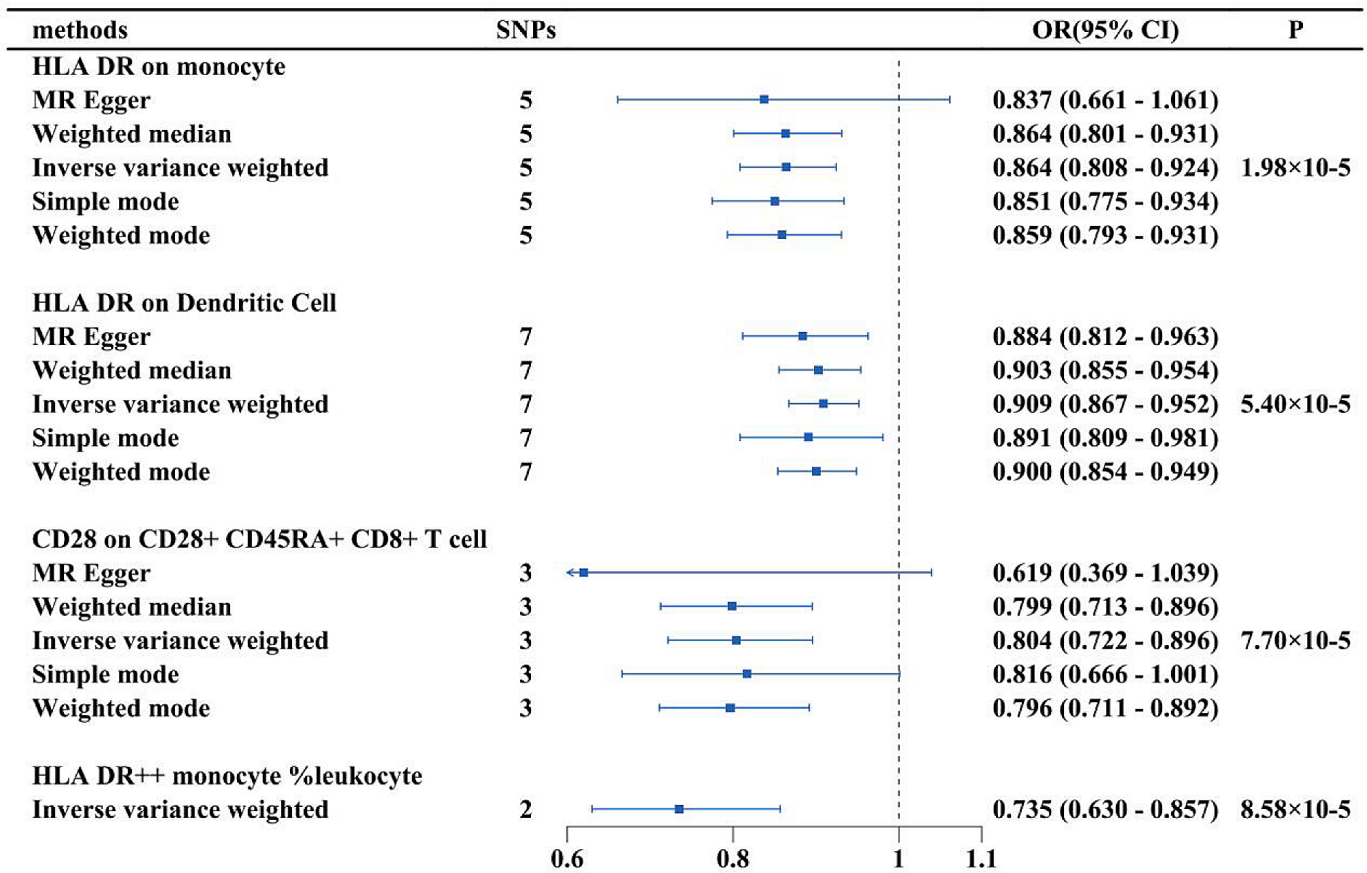
The forest plot shows the causal relationship between immune cell traits and AK

Our analysis identified potential causal relationships between four types of immune cells and AK. An increase in the abundance of HLA DR on monocytes, HLA DR on dendritic cells, CD28 on CD28 + CD45RA + CD8 + T cells, and HLA DR + + monocyte %leukocyte was negatively correlated with the risk of AK, indicating that an increase in the abundance of these immune cells leads to a decrease in AK risk. The IVW analysis results for all immune cells are as follows: HLA DR on monocyte (OR = 0.864, 95% CI = 0.808–0.924, *p* = 1.98E-04), HLA DR on dendritic cell (OR = 0.909, 95% CI = 0.867–0.952, *p* = 5.40E-04), CD28 on CD28 + CD45RA + CD8 + T cell (OR = 0.804, 95% CI = 0.722–0.896, *p* = 7.70E-04), HLA DR + + monocyte %leukocyte (OR = 0.735, 95% CI = 0.630–0.857, *p* = 8.58E-04). We performed reverse MR analysis of all immune cell phenotypes with AK, all MR results were greater than 0.05, indicating that AK had no impact on the included immune cells. The final results demonstrate potential causal relationships between the four immune cell phenotypes and AK (Fig. 5).

### Causal relationship between immune cells and SCC

In order to strictly control the deviation, the FDR was adjusted in this study, and only immune cells with PFDR less than 0.05 were considered to have a defined causal relationship with the outcome. Therefore, no immune cells were found to have a potential causal association with SCC in this study. Secondly, it may be related to the formation of an immunosuppressive microenvironment in which immune cell function is impaired. The latest research results have proved that although SCC is a highly immunogenic skin cancer, the abnormal epigenetic characteristics of tumor cells during its development not only affect the malignant phenotype of tumor cells, but also alter the function of immune cells and remodel the tumor microenvironment, which can diminish the efficacy of immune cells and the infiltration of immune cell [25]. In addition, tumor-derived function of tumor protein P63 (TP63), a master transcription factor in SCC, in promoting immune evasion and influencing the efficacy of immunotherapy in squamous carcinoma, revealing that IFNγ/α signaling is the pathway that is most significantly inhibited by TP63, which is usually specifically overexpressed in SCC, and that in human SCC patients, TP63 expression was negatively correlated with CD8 + T cell infiltration and activation [26]. However, since AK serves as a precursor lesion to SCC, further analysis of the potential causal relationships between immune cell subtypes and AK could provide new insights for the prevention and early treatment of SCC.

In the sensitivity analysis, we conducted heterogeneity and pleiotropy analyses for the immune cells included in the study and their corresponding skin cancer diseases. The results showed *p*-values greater than 0.05, indicating the absence of heterogeneity and pleiotropy in the SNPs (Tables 1 and 2). Additionally, leave-one-out analysis was performed, further confirming the stability of the causal relationships mentioned above. Scatter plots and funnel plots also indicated the stability of the results. The leave-one-out plot, scatter plots and funnel plots are available in the Supplementary Materials.

**Table 1:** The heterogeneity test of immune cells and skin cancer

exposure	outcome	method	Q	Q_df	Q_pval
CD25 on IgD + CD24- B cell	MM	MR Egger	0.03	1	0.86
CD25 on IgD + CD24- B cell	MM	IVW	0.03	2	0.98
CD25 on IgD- CD38dim B cell	BCC	MR Egger	0.45	1	0.50
CD25 on IgD- CD38dim B cell	BCC	IVW	1.96	2	0.38
CD33 on CD33 + HLA DR+	BCC	MR Egger	1.68	2	0.43
CD33 on CD33 + HLA DR+	BCC	IVW	2.33	3	0.51
CD33 on Monocytic Myeloid-Derived Suppressor Cells	BCC	MR Egger	1.68	2	0.43
CD33 on Monocytic Myeloid-Derived Suppressor Cells	BCC	IVW	2.34	3	0.51
CD33 on CD33 + HLA DR + CD14-	BCC	MR Egger	1.71	2	0.42
CD33 on CD33 + HLA DR + CD14-	BCC	IVW	2.37	3	0.50
CD33 on CD33 + HLA DR + CD14dim	BCC	MR Egger	1.68	2	0.43
CD33 on CD33 + HLA DR + CD14dim	BCC	IVW	2.38	3	0.50
HLA DR on CD33- HLA DR+	BCC	MR Egger	0.35	1	0.56
HLA DR on CD33- HLA DR+	BCC	IVW	2.86	2	0.24
Basophil %CD33dim HLA DR- CD66b-	BCC	MR Egger	2.01	1	0.16
Basophil %CD33dim HLA DR- CD66b-	BCC	IVW	2.56	2	0.28
Granulocytic Myeloid-Derived Suppressor Cells Absolute Count	BCC	IVW	0.17	1	0.68
HLA DR on monocyte	AK	MR Egger	3.93	3	0.27
HLA DR on monocyte	AK	IVW	4.03	4	0.40
HLA DR on Dendritic Cell	AK	MR Egger	3.79	5	0.58
HLA DR on Dendritic Cell	AK	IVW	4.35	6	0.63
CD28 on CD28 + CD45RA + CD8 + T cell	AK	MR Egger	0.01	1	0.98
CD28 on CD28 + CD45RA + CD8 + T cell	AK	IVW	1.02	2	0.60
HLA DR + + monocyte %leukocyte	AK	IVW	0.32	1	0.57

**Table 2:** Pleiotropy test for immune cells and skin cancer (some immune cells cannot be tested due to insufficient SNPs included)

exposure	outcome	egger_intercept	se	pval
CD25 on IgD + CD24- B cell	MM	0.00	0.00	1.00
CD25 on IgD- CD38dim B cell	BCC	-0.02	0.02	0.44
CD33 on CD33 + HLA DR+	BCC	0.01	0.01	0.50
CD33 on Monocytic Myeloid-Derived Suppressor Cells	BCC	0.01	0.01	0.50
CD33 on CD33 + HLA DR + CD14-	BCC	0.01	0.01	0.50
CD33 on CD33 + HLA DR + CD14dim	BCC	0.01	0.01	0.50
HLA DR on CD33- HLA DR+	BCC	0.03	0.02	0.36
Basophil %CD33dim HLA DR- CD66b-	BCC	0.02	0.03	0.70
HLA DR on monocyte	AK	0.01	0.05	0.80
HLA DR on Dendritic Cell	AK	0.02	0.02	0.49
CD28 on CD28 + CD45RA + CD8 + T cell	AK	0.15	0.14	0.50

## Discussion

The MR analysis elucidated potential causal relationships between exposure factors and outcomes. In this study, we used MR analysis to genetically explore the causal relationship between 731 immune cell phenotypes and skin cancer disease. This represents the first MR analysis investigating the causal relationships between various immune cell phenotypes and skin cancer diseases. Among the four immune traits (MFI, RC, AC, and MP), our analyses revealed that one type of immune cell was potentially causally associated with MM, eight types of immune cells were potentially causally associated with BCC, and four types of immune cells were potentially causally associated with AK.

In MM, the immune cell subtype associated with CD25 is closely linked to the disease. CD25 is the alpha chain of the heterotrimeric IL-2 receptor. In various hematologic malignancies, CD25 is highly expressed, but its expression levels are generally lower in most solid tumors [27]. CD25 is highly expressed on both resting and activated regulatory T (Tregs) cells but is not expressed on naïve T cells, memory T cells, or follicular helper T cells. Therefore, CD25 is currently the most commonly used marker for Tregs cells [28]. In 1995, Sakaguchi et al. [29]. first discovered that Tregs have immunosuppressive and immune-regulatory functions and reported that the Tregs phenotype is CD4 + and CD25+. CD25 is primarily involved in the differentiation and proliferation of regulatory CD4 + T cells. In CD4 + CD25 + Tregs cells, CD25 serves as a crucial component of the IL-2 receptor, inducing structural changes in IL-2, thereby promoting the formation of the IL2Rα/β/γ and IL-2 tetramer, activating JAK/STAT5, PI3K/Akt/mTOR, and mitogen-activated protein kinase (MAPK) signaling pathways, enabling Treg cells to exert immune-regulatory functions [30–32]. Experiments by Shang et al. demonstrated that Treg cell numbers significantly increase in different types of tumor microenvironments (TME), and the degree of Treg cell infiltration is associated with poor tumor prognosis [33]. Depletion of Tregs not only eliminates Treg-mediated immunosuppressive activity but also stimulates anti-tumor immunity. Rasku et al. demonstrated through a phase II trial in patients with unresectable stage IV melanoma that transient Treg clearance can reduce melanoma metastasis and promote the proliferation of tumor-specific effector T cells [34]. While CD25 is primarily expressed on activated T cells, it is also expressed in some B cell subgroups. On B cells, CD25 acts as an activation marker, indicating that B cells have been stimulated and have entered an activated state. Activated B cells can produce specific antibodies or IL-2. In the TME, they participate in M1 cell polarization and recruit effector T cells, playing an anti-cancer role [35]. The MR analysis in this study showed a negative correlation between CD25-labelled B cells and the risk of MM. However, research on the anti-cancer mechanisms of CD25-marked B cells is currently lacking. Therefore, the conclusions of this study provide a theoretical foundation for subsequent research.

HLA DR, which stands for Human Leukocyte Antigen - DR is a major histocompatibility complex (MHC) class II antigen expressed on the surface of B lymphocytes, monocytes, and macrophages, playing a role in presenting antigens to CD4 + T cells. The majority of T cells do not express HLA DR, and it is only in the late stages of T cell activation during the immune response that a proportion of activated T cells can express HLA DR. HLA DR is an important antigen on the surface of monocytes, and it is critical for the recognition of foreign antigens in the activation of specific T cells for the immune response. Kohchiyama et al. [36]. suggested that in BCC, the expression of the HLA DR antigen on tumor cells not only participates in various immune activities but may also be involved in cellular immune responses, acting as a defense mechanism against tumor cell proliferation. In this study, we also found that HLA DR-associated immune cells were negatively correlated with the risk of BCC, suggesting that HLA DR may play an anti-tumour role in BCC. HLA DR is considered essential for initiating autoimmune reactions, and low HLA DR expression may reduce CD4 T cell-mediated anti-tumor immunity [37]. Gadeyne [38] et al. found by a multi-omics approach that the microenvironment of HLA DR-positive melanoma regions was enriched with characteristic antigen-presenting cells, including dendritic cells and macrophages, and that cytotoxic T-cell depletion phenotypes were more prevalent in these regions. Meanwhile, these areas also exhibit enhanced signals related to interferon-gamma (IFNγ), leukocyte adhesion, and monocyte proliferation. The expression of cytokines associated with germinal center cells, such as CXCL12, CXCL13, and CCL19, is also increased in these regions. This suggests that HLA-DR-positive regions in melanoma attract anti-tumour immune cell infiltration by creating an atrophic microenvironment similar to the germinal centre. The findings indicate a complex interplay between HLA DR expression, immune cell composition, and the microenvironment in the context of Basal Cell Carcinoma, shedding light on the potential immunological mechanisms involved in this type of skin cancer. But as of yet, research on the role of HLA-DR-associated cells in BCC disease is limited, and the relationship between HLA DR and the immune system remains to be explored.

AK is a common precancerous skin lesion characterized by chronic reactive proliferation of keratinocytes, and some lesions may progress to SCC. Changes in immune defense mechanisms are believed to play a crucial role in the process of AK transforming into SCC. Hu et al. [39]. found a group of fibroblasts specifically expressing tryptophan 2,3-dioxygenase (TDO2) in the distant part of cancer nests, while CD4 + T cells and CD8 + T cells were enriched around these fibroblasts, demonstrating that TDO2 has a chemotactic capacity for T cells, inducing the transformation of CD4 + T cells into Treg cells, leading to functional impairment of CD8 + T cells. Treatment with a TDO2 inhibitor restored the anti-tumor function of T cells and prevented the malignant progression of AK to SCC. In MR analysis, we found that HLA DR and CD28-associated immune cells were negatively correlated with the risk of AK, so we suggest that altered immune defence mechanisms play an important role in the transformation of AK into SCC. Comparing with AK and early-stage SCC, there was a significant increase in the number of CD8 + T cells in advanced SCC. Activated HLA DR and IL-2R CD4 + T cells were able to directly kill tumor cells by mediating MHC II, inhibiting tumor cell growth [40]. In primary SCC, CD8 + T cells exhibited high expression of CTLA4 and TIGIT, while in recurrent SCC, CD8 + T cells showed high expression of HAVCR2 and CXCL13, down-regulating TNF and IL-17 signaling pathways and up-regulating oxidative phosphorylation functions. This indicated that T cell exhaustion in primary and recurrent SCC is caused by different immune inhibitory factors, and T cells in recurrent SCC are in a high metabolic state [41]. Similarly, reducing macrophage-mediated T cell rejection can enhance the surveillance of CD8 + T cells against tumors [42]. CD28 is a co-stimulatory molecule expressed on the surface of T lymphocytes, playing a crucial role in T cell activation. It binds to B7 molecules on antigen-presenting cells (APCs), mediating T cell co-stimulation and promoting T cell survival, proliferation, and cytokine production [43]. In addition, high levels of CD28 signaling can enhance the glycolytic pathway, further promoting the differentiation of exhausted precursor T cells (Tpex) into exhausted terminal T cells (Tex) [44]. Currently, immune checkpoint inhibitors (ICIs) have revolutionized tumour therapy, such as monacizumab targeting NKG2A inhibition to enhance NK cell and CD8 + T cell activity and further promote anti-tumor immunity [45], but ICIs still present a number of challenges. One of the biggest challenges is that only a small number of patients are able to benefit from current immunotherapies, and many patients develop resistance to treatment or fail to produce the expected response. Recently investigators identified Immunoscore-IC, a powerful biomarker that predicts the effectiveness of (ICIs) in tumor patients. Immunoscore-IC quantifies the density of CD4 + T and CD8 + cells and the distance between their cells in the tumor microenvironment, distinguishes between patients with tumors that respond and those that do not respond to treatment with ICIs, and is considered a promising predictive marker of response to antimmunotherapy [46, 47]. Mlecnik B [48] et al. found that Immunoscore accurately stratified high-risk and low-risk patients and acted as a predictor of response to chemotherapy.

After the first development of tumor immunotherapies, the understanding of tumor resistance and immunosuppression has gradually deepened, and the demand for personalized and precise medicine has gradually increased, and immunotherapy has become a major breakthrough in the field of cancer treatment [49]. However, despite the impressive achievements of immunotherapies such as immune checkpoint inhibitors and CAR-T cell therapy, only a small number of patients are still able to benefit from current immunotherapies [50], and many patients become resistant to treatment or fail to produce the expected response, which can be partly explained by differences in immune subtypes. Therefore, by comprehensively analyzing the immune subtypes of tumors, gaining a deeper understanding of the interactions between tumors and the immune system, and exploring the molecular mechanisms of the immune response, we can more accurately predict the response of patients to specific immune therapies, and thus guide a more personalized therapeutic strategy.

Second, immune subtyping provides a theoretical basis for precision immunotherapy. With the advent of the era of precision medicine, the concept of immune subtyping provides a new personalized dimension for cancer treatment. By gaining a deeper understanding of the complexity of the tumor immune microenvironment and how it affects the therapeutic effects, immune subtyping is expected to become an important indicator for assessing patients’ suitability for specific immunotherapy regimens. This study reveals the causal relationship between different types of immune cell subtypes and four types of skin cancers through an in-depth analysis of genetic data from different types of skin cancers. These subtypes span different cancer types, revealing commonalities and differences in tumor immune responses. This finding not only provides a new perspective for understanding tumor immune escape mechanisms, but also provides a theoretical basis for precision immunotherapy.

In addition, immune subtypes serve as potential indicators for personalized medicine. The analysis of immune subtypes can also reveal potential therapeutic targets and provide clues for the development of new immunotherapeutic strategies. For example, certain immune subtypes may indicate that a tumor is particularly sensitive to specific immunomodulatory molecules [51], and these molecules can then become new therapeutic targets. Similarly, by analyzing the immune subtypes of patients whose response is predicted to be poor, the possibility of using other treatments in combination to improve the therapeutic outcome can be explored. Therefore, this study delves into the relationship between tumor immune subtypes and different types of skin cancer, providing an important biological basis for personalized medicine.

Finally, this study reveals the causal relationship between specific immune subtypes and different types of skin cancer, providing clues for the discovery of new therapeutic targets and drugs. The application of this statistical approach based on genetic variation not only enhances our understanding of the tumor immune microenvironment, but also offers the possibility of developing new immunotherapeutic strategies.

This study has several strengths and limitations. Firstly, the research employed a large sample size for immune traits and skin cancer, enhancing statistical efficiency in the two-sample Mendelian randomization (MR) analysis. Secondly, the conclusions drawn in this study are based on genetic instrumental variables, utilizing five MR analysis methods for causal inference, providing results with a degree of stability and less susceptibility to horizontal pleiotropy and heterogeneity. However, the study has certain limitations. First, due to a lack of individual-level information, further stratified analysis within the general population was not feasible. Second, immune cell data and skin cancer data were derived from different studies, introducing some differences in sample size, quality control methods, and racial composition, which might lead to some errors. Third, the majority of participants in the GWAS data used in this study were of European ancestry, potentially impacting the generalizability of the findings to other ethnic groups.

## Conclusion

In summary, we demonstrated a causal relationship between immune cells and skin cancer by comprehensive bidirectional MR analysis. In addition, our study significantly reduced the effects of unavoidable confounders, reverse causality, and other factors. This may provide new insights for researchers to explore the immunology of skin cancer pathogenesis and help to explore early intervention and therapeutic approaches. However, there are some limitations of this study and more experimental studies are still needed to further explore the potential mechanisms between the identified immune cells and skin cancer risk.

### Electronic supplementary material

Below is the link to the electronic supplementary material.

Supplementary Material 1

Supplementary Material 2

Supplementary Material 3

Supplementary Material 4

Supplementary Material 5

## Data Availability

The data used in the present study are all publicly available at https://gwas.mrcieu.ac.uk/. The original contributions presented in the study are included in the article/Supplementary Material.

## Abbreviations

MM: Malignant melanoma
BCC: Basal cell carcinoma
SCC: Squamous cell carcinoma
AK: Actinic keratosis
MR: Mendelian randomization
IV: Instrumental variable
APC: Antigen-presenting cells
TME: Tumor microenvironment
Tregs: Regulatory T cells
GWAS: Genome-wide association studies
MFI: Median fluorescence intensities
RC: Relative cell
AC: Absolute cell
MP: Morphological parameters
SNP: Single nucleotide polymorphism
FDR: False discovery rate
