# Supplementary figures and images for: Causal association between skin cancer and immune cells: mendelian randomization (MR) study
Source: BMC Cancer. 2024 Jul 17;24:849. doi: 10.1186/s12885-024-12603-0 (PMC11256556; doi:10.1186/s12885-024-12603-0)

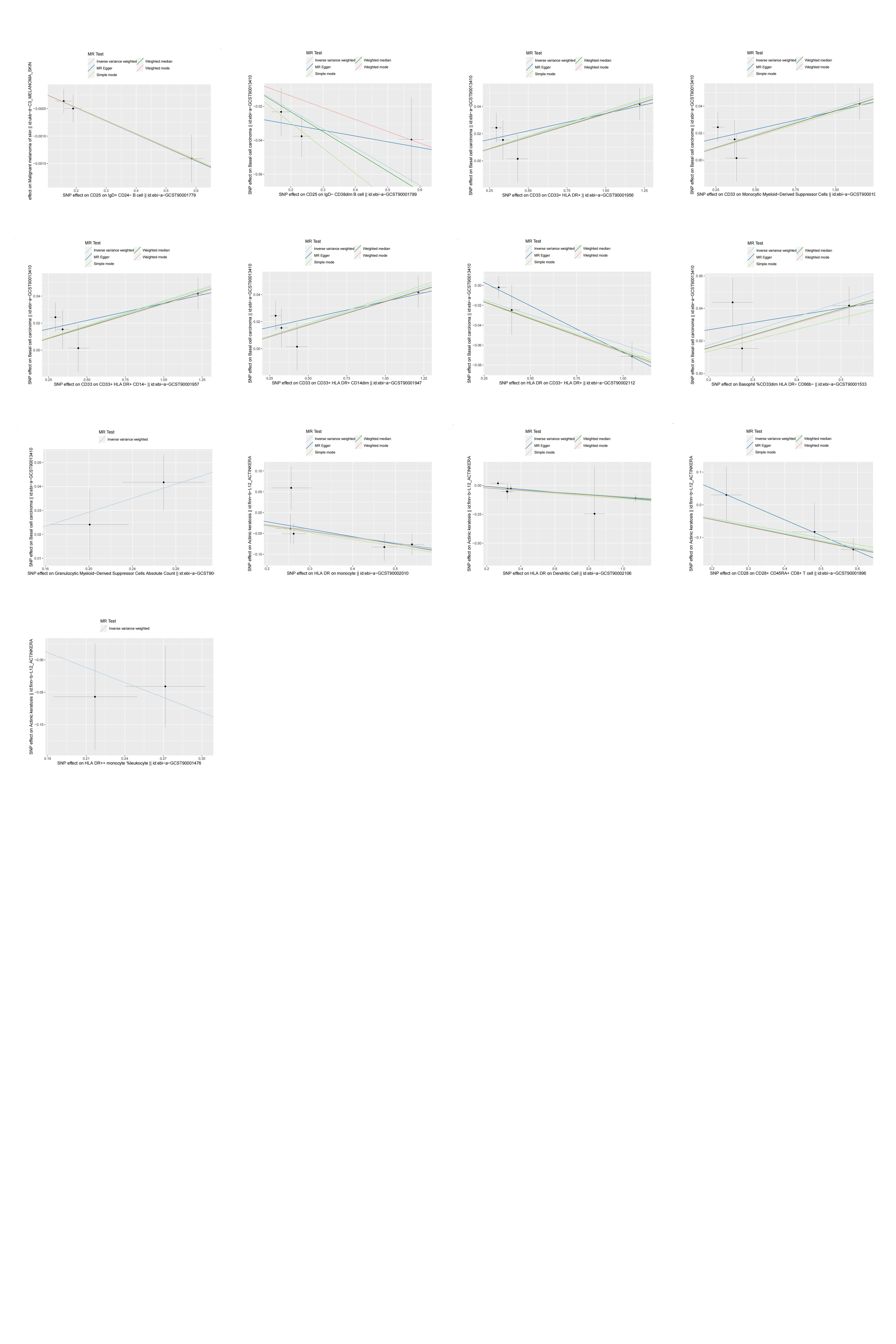

Supplement: Supplementary file 2 — Supplementary Material 2 [file 12885_2024_12603_MOESM2_ESM.png]

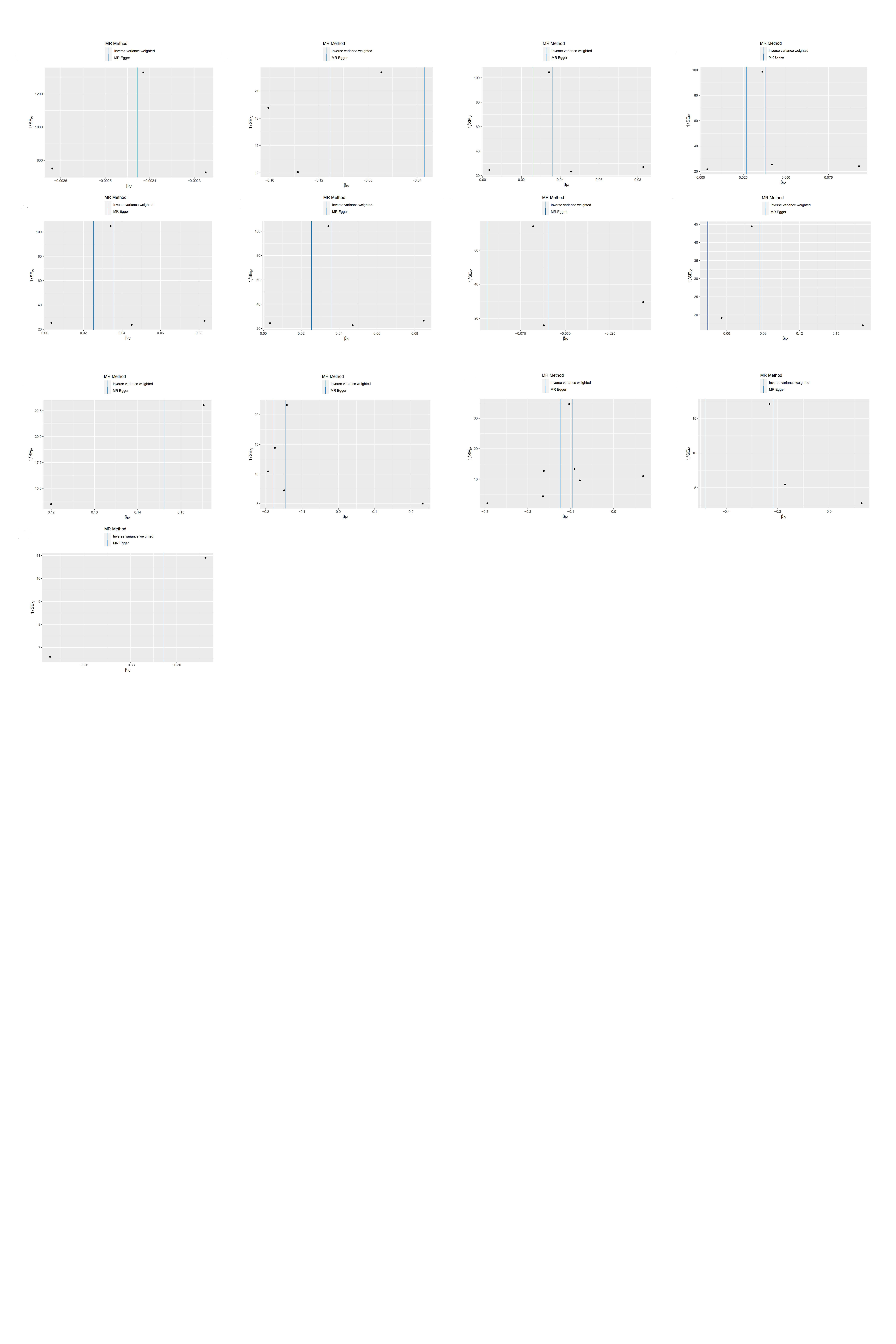

Supplement: Supplementary file 3 — Supplementary Material 3 [file 12885_2024_12603_MOESM3_ESM.png]
