# Supplementary material for: Causal association between skin cancer and immune cells: mendelian randomization (MR) study
Source: BMC Cancer. 2024 Jul 17;24:849. doi: 10.1186/s12885-024-12603-0 (PMC11256556; doi:10.1186/s12885-024-12603-0)
Supplement: Supplementary file 5 — Supplementary Material 5 [file 12885_2024_12603_MOESM5_ESM.pdf]

STROBE-MR checklist of recommended items to address in reports of Mendelian randomization studies[1 2]

Item No.	Section	Checklist item	Page No.	Relevant text from manuscript
1	**TITLE and ABSTRACT**	Indicate Mendelian randomization (MR) as the study's design in the title and/or the abstract if that is a main purpose of the study	1	Title<br>"Causal Association between Skin Cancer and Immune Cells: Mendelian randomization (MR) study".<br>Abstract<br>"Background: Numerous meta-analyses and clinical studies have shown that subtypes of immune cells are associated with the development of skin cancer, but it is not clear whether this association is causal or biased. Mendelian randomization (MR) analysis reduces the effect of confounding factors and improves the accuracy of the results when compared to traditional studies. Thus, in order to examine the causal relationship between various immune cell and skin cancer, this study employs two-sample MR.<br>Methods: This study assesses the causal association between 731 immune cell characteristics and skin cancer using a two-sample Mendel randomization (MR) methodology. Multiple MR methods were used to bias and to derive reliable estimates of causality between instrumental variables and outcomes. Comprehensive sensitivity analyses were used to validate the stability, heterogeneity and horizontal multiplicity of the results.<br>Results: We discovered that potential causal relationships between different types of immune cells and skin cancer disease. Specifically, one type of immune cell as potentially causal to malignant melanoma of skin (MM), eight different types of immune cells as potentially causal to basal cell carcinoma (BCC), four different types of immune cells as potentially causal to actinic keratosis (AK), and no different types of immune cells were found to have a potential causal association with squamous cell carcinoma （SCC）, with stability in all of the results.<br>Conclusion: This study demonstrates the close

				connection between immune cells and skin cancer disease by genetic means, which enriches the current knowledge about the role of immune cells in skin cancer and also contributes to the design of therapeutic strategies from an immunological perspective.”
	**INTRODUCTION**			
2	**Background**	Explain the scientific background and rationale for the reported study. What is the exposure? Is a potential causal relationship between exposure and outcome plausible? Justify why MR is a helpful method to address the study question	1	Multiple relevant studies have indicated that the immune system plays a pivotal role in the occurrence, growth, and metastasis of malignancies, but it is not clear whether this association is causal or biased. In Mendelian randomisation (MR), genetic variants are used as instrumental variables to test for a causal association between a risk factor and an outcome. MR analysis reduces the effect of confounding factors and improves the accuracy of the results when compared to traditional studies.
3	**Objectives**	State specific objectives clearly, including pre-specified causal hypotheses (if any). State that MR is a method that, under specific assumptions, intends to estimate causal effects	2	This study utilized the MR method to analyze the causal relationship between different types of immune cell phenotypes and skin cancer in the European population.
	**METHODS**			
4	**Study design and data sources**	Present key elements of the study design early in the article. Consider including a table listing sources of data for all phases of the study. For each data source contributing to the analysis, describe the following:	2-3	Details of the contributing GWAS consortiums are listed in supplementary materials. The studies were selected for investigating traits related to skin cancer or immune cells, having the largest sample sizes, and consisting of the most similar populations while minimising sample overlap.
	a)	Setting: Describe the study design and the underlying population, if possible. Describe the setting, locations, and relevant dates, including periods of recruitment, exposure, follow-up, and data collection, when available.	2-3	The SardiNIA project is a longitudinal study comprising 6,602 general population individuals (57% females, 43% males), ranging from 18 to 102 years, native of the central east coast of Sardinia, Italy. All volunteers are deeply genetically characterized and 3,757 of them are immune profiled. Researcher estimated the heritability for all inverse-normalized traits in cohort of 3,757 immunophenotyped individuals. Heritability estimation exploit the unique features of our cohort, where 3,371 samples among the 3,757 are grouped in 847 multigenerational families (from 1 to 5 generations, average 2.66), comprising 2,405

			sib-pairs (including 4 monozygotic twins), 79 half-sib pairs, 2,258 cousins-pairs, 1,587 parent-child pairs, 88 grandparent-grandchild pairs and 2,997 avuncular pairs.
b)	Participants: Give the eligibility criteria, and the sources and methods of selection of participants. Report the sample size, and whether any power or sample size calculations were carried out prior to the main analysis	2-3	The SardiNIA project is a longitudinal study comprising 6,602 general population individuals (57% females, 43% males), ranging from 18 to 102 years, native of the central east coast of Sardinia, Italy. Researchers report on the influence of ~22 million variants on 731 immune cell traits in a cohort of 3,757 Sardinians. We detected 122 significant ($P<1.28 \times 10^{-11}$) independent association signals for 459 cell traits at 70 loci (53 of them novel) identifying several molecules and mechanisms involved in cell regulation.
c)	Describe measurement, quality control and selection of genetic variants	2-3	To identify SNPs associated with the exposure factor and ensure the reliability and accuracy of conclusions regarding the relationship between immune cells and skin cancer risk, the following steps were taken to select the most optimal SNPs. Initially, acknowledging that only a limited number of SNPs in the immune cell group met the genome-wide significance threshold ($p<5\times10^{-8}$). Additionally, to ensure the independence of the selected instrumental variables (IVs) and minimize bias resulting from residual linkage disequilibrium of genetic variations, we employed the two-sample MR R package with a distance set at 10,000 kb and a linkage disequilibrium threshold of $r^2<0.001$. On top of that, to mitigate the potential bias resulting from weak instrumental variables, the F-statistic was utilized to assess the statistical strength of the correlation between each SNP and the exposure. IVs with an F-statistic exceeding 10 were considered strong instruments, while those with an F-statistic less than 10 indicated a weak correlation between SNPs and the exposure. During the analysis, SNPs with palindromic structures were automatically excluded.
d)	For each exposure, outcome, and other relevant variables, describe methods of assessment and diagnostic criteria for diseases	2-3	In this study, we applied five MR methods to validate the causal associations between genetic variations in immune cells and MM, BCC, SCC, and AK. The methods comprised inverse variance weighted analysis (IVW), weighted median (WM), simple median (SM), weighted median estimator

					(WME), and MR-Egger regression. In the analysis, the IVW method, based on genotype summary data, served as the primary approach. The IVW method combined Wald estimates for each single nucleotide polymorphism through a meta-analysis, resulting in an overall estimate. The weighted regression slope of the effect of the result on the effect of the exposure, with the intercept constrained to zero, represented the overall estimate. This approach provided a comprehensive evaluation of the causal relationship between genetic variations in immune cells and MM, BCC, SCC, and AK.<br><br>In secondary sensitivity analysis, we employed Cochran's Q method to assess the heterogeneity of the selected IVs. A significant result (p<0.05) would indicate significant heterogeneity in the analysis outcomes. To reduce the influence of horizontal pleiotropy, we utilized the MR-Egger regression test. If a significant intercept term is observed (p<0.05), it suggests the presence of horizontal pleiotropy. Additionally, we applied the Bonferroni method for correction, considering only results with p-values < Bonferroni value in the final analysis. The Bonferroni correction formula is 0.05 / (number of exposures included in the study * number of outcomes included in the study).<br><br>Finally, in order to explore whether skin cancer diseases have any causal relationship with established important immune cells, we conducted a reverse MR analysis using SNPs related to skin cancer diseases as IVs (skin cancer diseases as exposure and established immune cells as outcomes).
		e)	Provide details of ethics committee approval and participant informed consent, if relevant	8	Informed consent was obtained from all participants, and study protocols were approved by the local, regional, or institutional ethics committees.
5	**Assumptions**		Explicitly state the three core IV assumptions for the main analysis (relevance, independence and exclusion restriction) as well assumptions for any additional or sensitivity analysis	2-3	Mendelian randomization relies on three core assumptions: (1) genetic variation is directly associated with the exposure; (2) genetic variation is not associated with confounding factors between the exposure and the outcome; (3) genetic variation does not affect the outcome through mechanisms other than the exposure. In secondary

					sensitivity analysis, we employed Cochran's Q method to assess the heterogeneity of the selected IVs. A significant result ($p<0.05$) would indicate significant heterogeneity in the analysis outcomes. To reduce the influence of horizontal pleiotropy, we utilized the MR-Egger regression test. If a significant intercept term is observed ($p<0.05$), it suggests the presence of horizontal pleiotropy. Additionally, we applied the Bonferroni method for correction, considering only results with p-values < Bonferroni value in the final analysis. The Bonferroni correction formula is 0.05 / (number of exposures included in the study * number of outcomes included in the study).
6	**Statistical methods: main analysis**		Describe statistical methods and statistics used		
		a)	Describe how quantitative variables were handled in the analyses (i.e., scale, units, model)	3	In this study, we applied five MR methods to validate the causal associations between genetic variations in immune cells and MM, BCC, SCC, and AK. The methods comprised inverse variance weighted analysis (IVW), weighted median (WM), simple median (SM), weighted median estimator (WME), and MR-Egger regression. In the analysis, the IVW method, based on genotype summary data, served as the primary approach. The IVW method combined Wald estimates for each single nucleotide polymorphism through a meta-analysis, resulting in an overall estimate. The weighted regression slope of the effect of the result on the effect of the exposure, with the intercept constrained to zero, represented the overall estimate. The statistics of the above five methods include p-values and OR values. When the p-value of the MR result is less than 0.05, it indicates an association between the exposure and the outcome. When the OR value is greater than 1, it signifies a positive association between the exposure and the outcome, meaning that an increase in the exposure factor leads to an increase in the risk factor of the outcome, and suggesting that the exposure may be a risk factor for the outcome. When the OR value is less than 1, it indicates a negative association between the exposure and the outcome, meaning that an increase in the exposure factor leads to a decrease

					in the risk factor of the outcome, and suggesting that the exposure may be a protective factor for the outcome. By employing the above five MR methods, we aimed to minimize bias and obtain reliable estimates of the causal relationship between the exposure of interest and the outcome.
		b)	Describe how genetic variants were handled in the analyses and, if applicable, how their weights were selected	3	When the OR value is greater than 1, it signifies a positive association between the exposure and the outcome, meaning that an increase in the exposure factor leads to an increase in the risk factor of the outcome, and suggesting that the exposure may be a risk factor for the outcome. When the OR value is less than 1, it indicates a negative association between the exposure and the outcome, meaning that an increase in the exposure factor leads to a decrease in the risk factor of the outcome, and suggesting that the exposure may be a protective factor for the outcome.
		c)	Describe the MR estimator (e.g. two-stage least squares, Wald ratio) and related statistics. Detail the included covariates and, in case of two-sample MR, whether the same covariate set was used for adjustment in the two samples	3	We obtained SNP-specific Wald estimates and then meta-analysed them using inverse variance weighting (IVW) with multiplicative random effects.
		d)	Explain how missing data were addressed	2-3	There were no missing data in this study.
		e)	If applicable, indicate how multiple testing was addressed	3	To avoid bias from weak instrumental variables, we used the F-statistic to assess the statistical strength of the correlation between each SNP and the exposures. IVs with an F-statistic > 10 were considered strong instruments, while those with F < 10 were deemed to have a weak correlation between SNPs and the exposures. During each analysis, SNPs with palindromic structures were automatically excluded. Additionally, we applied the Bonferroni method for correction, considering only results with p-values < Bonferroni value in the final analysis. The Bonferroni correction formula is 0.05 / (number of exposures included in the study * number of outcomes included in the study).
7	**Assessment of assumptions**		Describe any methods or prior knowledge used to assess the assumptions or justify their validity	3	We restricted the inclusion criteria for IVs to ensure the accuracy and effectiveness of the causal relationship between immune cells and the risk of skin cancer. Firstly, only SNPs with a P-value <5e-08 were included as exposure and outcome IVs in the MR study. Secondly, the Two Sample MR R package was used with the settings of r2 = 0.001

and kb=10000 to ensure the independence of the selected IVs and minimize violation of the random allele distribution resulted from linkage disequilibrium effects, only SNPs that meet the p-value criteria and have been cleared of linkage disequilibrium are eligible to match with exposure. In addition, to avoid bias from weak instrumental variables, we used the F-statistic to assess the statistical strength of the correlation between each SNP and the exposures. IVs with an F-statistic > 10 were considered strong instruments, while those with F< 10 were deemed to have a weak correlation between SNPs and the exposures. During each analysis, SNPs with palindromic structures were automatically excluded. We excluded SNPs with an F-statistic value less than 10, as an Fstatistic value greater than 10 indicates sufficient strength to ensure the validity of the SNPs. And we applied five MR methods to validate the causal associations between genetic variations in immune cells and MM, BCC, SCC, and AK. The methods comprised inverse variance weighted analysis (IVW), weighted median (WM), simple median (SM), weighted median estimator (WME), and MR-Egger regression. In the analysis, the IVW method, based on genotype summary data, served as the primary approach. The IVW method combined Wald estimates for each single nucleotide polymorphism through a meta-analysis, resulting in an overall estimate. The weighted regression slope of the effect of the result on the effect of the exposure, with the intercept constrained to zero, represented the overall estimate. This approach provided a comprehensive evaluation of the causal relationship between genetic variations in immune cells and MM, BCC, SCC, and AK. In secondary sensitivity analysis, we employed Cochran's Q method to assess the heterogeneity of the selected IVs. A significant result (p<0.05) would indicate significant heterogeneity in the analysis outcomes. To reduce the influence of horizontal pleiotropy, we utilized the MR-Egger regression test. If a significant intercept term is observed (p<0.05), it suggests the presence of horizontal pleiotropy. Additionally, we applied the Bonferroni method for correction, considering only results with p-values < Bonferroni value in the final analysis. The Bonferroni

				correction formula is 0.05 / (number of exposures included in the study * number of outcomes included in the study).
8	**Sensitivity analyses and additional analyses**	Describe any sensitivity analyses or additional analyses performed (e.g. comparison of effect estimates from different approaches, independent replication, bias analytic techniques, validation of instruments, simulations)	3	To identify SNPs associated with the exposure factor and ensure the reliability and accuracy of conclusions regarding the relationship between immune cells and skin cancer risk, the following steps were taken to select the most optimal SNPs. Initially, acknowledging that only a limited number of SNPs in the immune cell group met the genome-wide significance threshold ($p<5\times10^{-8}$). Additionally, to ensure the independence of the selected instrumental variables (IVs) and minimize bias resulting from residual linkage disequilibrium of genetic variations, we employed the two-sample MR R package with a distance set at 10,000 kb and a linkage disequilibrium threshold of $r^2<0.001$. On top of that, to mitigate the potential bias resulting from weak instrumental variables, the F-statistic was utilized to assess the statistical strength of the correlation between each SNP and the exposure. IVs with an F-statistic exceeding 10 were considered strong instruments, while those with an F-statistic less than 10 indicated a weak correlation between SNPs and the exposure. During the analysis, SNPs with palindromic structures were automatically excluded.<br><br>And we applied five MR methods to validate the causal associations between genetic variations in immune cells and MM, BCC, SCC, and AK. The methods comprised inverse variance weighted analysis (IVW), weighted median (WM), simple median (SM), weighted median estimator (WME), and MR-Egger regression. In the analysis, the IVW method, based on genotype summary data, served as the primary approach. The IVW method combined Wald estimates for each single nucleotide polymorphism through a meta-analysis, resulting in an overall estimate. The weighted regression slope of the effect of the result on the effect of the exposure, with the intercept constrained to zero, represented the overall estimate. This approach provided a comprehensive evaluation of the causal relationship between genetic variations in immune cells and MM, BCC, SCC, and AK.

					In secondary sensitivity analysis, we employed Cochran's Q method to assess the heterogeneity of the selected IVs. A significant result (p<0.05) would indicate significant heterogeneity in the analysis outcomes. To reduce the influence of horizontal pleiotropy, we utilized the MR-Egger regression test. If a significant intercept term is observed (p<0.05), it suggests the presence of horizontal pleiotropy. Additionally, we applied the Bonferroni method for correction, considering only results with p-values < Bonferroni value in the final analysis. The Bonferroni correction formula is 0.05 / (number of exposures included in the study * number of outcomes included in the study).<br>Finally, in order to explore whether skin cancer diseases have any causal relationship with established important immune cells, we conducted a reverse MR analysis using SNPs related to skin cancer diseases as IVs (skin cancer diseases as exposure and established immune cells as outcomes).
9	**Software and pre-registration**				
		a)	Name statistical software and package(s), including version and settings used	3	All statistical analyses were implemented by the package Two-Sample MR (version 0.5.6) and Radial MR (version 1.0) in R (version 4.2.1).
		b)	State whether the study protocol and details were pre-registered (as well as when and where)	2	Using a two-sample Mendelian randomization (MR) approach, we conducted an analysis to assess the causal relationships between 731 immune cell subtypes and skin cancer. In the study design, we employed various MR methods to minimize bias and obtain reliable estimates of the modifiable exposures of interest and their relationship with the outcomes.
	**RESULTS**				
10	**Descriptive data**				
		a)	Report the numbers of individuals at each stage of included studies and reasons for exclusion. Consider use of a flow diagram	2-3	To identify SNPs associated with the exposure factor and ensure the reliability and accuracy of conclusions regarding the relationship between immune cells and skin cancer risk, the following steps were taken to select the most optimal SNPs. Initially, acknowledging that only a limited number

of SNPs in the immune cell group met the genome-wide significance threshold ($p<5\times10^{-8}$). Additionally, to ensure the independence of the selected instrumental variables (IVs) and minimize bias resulting from residual linkage disequilibrium of genetic variations, we employed the two-sample MR R package with a distance set at 10,000 kb and a linkage disequilibrium threshold of $r^2<0.001$. On top of that, to mitigate the potential bias resulting from weak instrumental variables, the F-statistic was utilized to assess the statistical strength of the correlation between each SNP and the exposure. IVs with an F-statistic exceeding 10 were considered strong instruments, while those with an F-statistic less than 10 indicated a weak correlation between SNPs and the exposure. During the analysis, SNPs with palindromic structures were automatically excluded. we applied five MR methods to validate the causal associations between genetic variations in immune cells and MM, BCC, SCC, and AK. The methods comprised inverse variance weighted analysis (IVW), weighted median (WM), simple median (SM), weighted median estimator (WME), and MR-Egger regression. In the analysis, the IVW method, based on genotype summary data, served as the primary approach. The IVW method combined Wald estimates for each single nucleotide polymorphism through a meta-analysis, resulting in an overall estimate. The weighted regression slope of the effect of the result on the effect of the exposure, with the intercept constrained to zero, represented the overall estimate. This approach provided a comprehensive evaluation of the causal relationship between genetic variations in immune cells and MM, BCC, SCC, and AK. In secondary sensitivity analysis, we employed Cochran's Q method to assess the heterogeneity of the selected IVs. A significant result ($p<0.05$) would indicate significant heterogeneity in the analysis outcomes. To reduce the influence of horizontal pleiotropy, we utilized the MR-Egger regression test. If a significant intercept term is observed ($p<0.05$), it suggests the presence of horizontal pleiotropy. Additionally, we applied the Bonferroni method for correction, considering only results with p-values < Bonferroni value in the final analysis. The Bonferroni correction formula is 0.05 / (number of

				exposures included in the study * number of outcomes included in the study). Finally, in order to explore whether skin cancer diseases have any causal relationship with established important immune cells, we conducted a reverse MR analysis using SNPs related to skin cancer diseases as IVs (skin cancer diseases as exposure and established immune cells as outcomes). The workflow is shown in Figure 2.
		b) Report summary statistics for phenotypic exposure(s), outcome(s), and other relevant variables (e.g. means, SDs, proportions)		Summary statistics of exposures and outcomes are provided in the supplementary materials, figure 3, figure 4 and figure 5.
		c) If the data sources include meta-analyses of previous studies, provide the assessments of heterogeneity across these studies	12	The heterogeneity test of immune cells and skin cancer is shown in Table 1.
		d) For two-sample MR:<br>i. Provide justification of the similarity of the genetic variant-exposure associations between the exposure and outcome samples<br>ii. Provide information on the number of individuals who overlap between the exposure and outcome studies	2-3	In this research, we obtained publicly available GWAS data for immune cells from the GWAS catalog and conducted an extensive analysis of genetic variations in a population of 3,757 individuals from Sardinia, evaluating 731 immune cell types to identify genetic variations associated with immune cell characteristics. Data on genetic variants in skin cancers were obtained from GWAS in Europeans.
11	**Main results**			
		a) Report the associations between genetic variant and exposure, and between genetic variant and outcome, preferably on an interpretable scale	4	An increase in the abundance of CD25 on IgD+ CD24- B cells was negatively correlated with the risk of MM (OR=0.998, 95% CI=0.996-1.000, p=4.04E-05) （Figure 3）.<br>An increase in the abundance of CD25 on IgD- CD38dim B cells and HLA DR on CD33- HLA DR+ was negatively correlated with the risk of BCC, while the remaining immune cells showed a positive correlation. The IVW analysis results are as follows: CD25 on IgD- CD38dim B cell (OR=0.895, 95% CI=0.842-0.951, p=3.37E-04), CD33 on CD33+ HLA DR+ (OR=1.037, 95% CI=1.019-1.055, p=4.53E-05), CD33 on Monocytic Myeloid-Derived Suppressor Cells (OR=1.039, 95% CI=1.020-1.059, p=4.54E-05), CD33 on CD33+ HLA DR+ CD14- (OR=1.036, 95% CI=1.019-1.055, p=4.63E-05), CD33 on CD33+ HLA DR+ CD14dim (OR=1.037, 95% CI=1.019-1.055, p=4.64E-05), HLA DR on CD33- HLA DR+

			(OR=0.942, 95% CI=0.915-0.970, p=5.01E-05), Basophil %CD33dim HLA DR- CD66b- (OR=1.091, 95% CI=1.045-1.140, p=7.49E-05), Granulocytic Myeloid-Derived Suppressor Cells Absolute Count (OR=1.158, 95% CI=1.076-1.255, p=9.48E-05) . (Figure 4).<br><br>An increase in the abundance of HLA DR on monocytes, HLA DR on dendritic cells, CD28 on CD28+ CD45RA+ CD8+ T cells, and HLA DR++ monocyte %leukocyte was negatively correlated with the risk of AK, indicating that an increase in the abundance of these immune cells leads to a decrease in AK risk. The IVW analysis results for all immune cells are as follows: HLA DR on monocyte (OR=0.864, 95% CI=0.808-0.924, p=1.98E-04), HLA DR on dendritic cell (OR=0.909, 95% CI=0.867-0.952, p=5.40E-04), CD28 on CD28+ CD45RA+ CD8+ T cell (OR=0.804, 95% CI=0.722-0.896, p=7.70E-04), HLA DR++ monocyte %leukocyte (OR=0.735, 95% CI=0.630-0.857, p=8.58E-04)（Figure 5）.
b)	Report MR estimates of the relationship between exposure and outcome, and the measures of uncertainty from the MR analysis, on an interpretable scale, such as odds ratio or relative risk per SD difference	4	An increase in the abundance of CD25 on IgD+ CD24- B cells was negatively correlated with the risk of MM (OR=0.998, 95% CI=0.996-1.000) .<br><br>In BCC, CD25 on IgD- CD38dim B cell (OR=0.895, 95% CI=0.842-0.951), CD33 on CD33+ HLA DR+ (OR=1.037, 95% CI=1.019-1.055), CD33 on Monocytic Myeloid-Derived Suppressor Cells (OR=1.039, 95% CI=1.020-1.059), CD33 on CD33+ HLA DR+ CD14- (OR=1.036, 95% CI=1.019-1.055), CD33 on CD33+ HLA DR+ CD14dim (OR=1.037, 95% CI=1.019-1.055), HLA DR on CD33- HLA DR+ (OR=0.942, 95% CI=0.915-0.970), Basophil %CD33dim HLA DR- CD66b- (OR=1.091, 95% CI=1.045-1.140), Granulocytic Myeloid-Derived Suppressor Cells Absolute Count (OR=1.158, 95% CI=1.076-1.255).<br><br>An increase in the abundance of HLA DR on monocytes, HLA DR on dendritic cells, CD28 on CD28+ CD45RA+ CD8+ T cells, and HLA DR++ monocyte %leukocyte was negatively correlated with the risk of AK, indicating that an increase in the abundance of these immune cells leads to a decrease in AK risk. HLA DR on monocyte (OR=0.864, 95% CI=0.808-0.924), HLA DR on

					dendritic cell (OR=0.909, 95% CI=0.867-0.952), CD28 on CD28+ CD45RA+ CD8+ T cell (OR=0.804, 95% CI=0.722-0.896), HLA DR++ monocyte %leukocyte (OR=0.735, 95% CI=0.630-0.857).
		c)	If relevant, consider translating estimates of relative risk into absolute risk for a meaningful time period	4	An increase in the abundance of CD25 on IgD+ CD24- B cells was negatively correlated with the risk of MM (OR=0.998, 95% CI=0.996-1.000) .<br>In BCC, CD25 on IgD- CD38dim B cell (OR=0.895, 95% CI=0.842-0.951), CD33 on CD33+ HLA DR+ (OR=1.037, 95% CI=1.019-1.055), CD33 on Monocytic Myeloid-Derived Suppressor Cells (OR=1.039, 95% CI=1.020-1.059), CD33 on CD33+ HLA DR+ CD14- (OR=1.036, 95% CI=1.019-1.055), CD33 on CD33+ HLA DR+ CD14dim (OR=1.037, 95% CI=1.019-1.055), HLA DR on CD33- HLA DR+ (OR=0.942, 95% CI=0.915-0.970), Basophil %CD33dim HLA DR- CD66b- (OR=1.091, 95% CI=1.045-1.140), Granulocytic Myeloid-Derived Suppressor Cells Absolute Count (OR=1.158, 95% CI=1.076-1.255).<br>An increase in the abundance of HLA DR on monocytes, HLA DR on dendritic cells, CD28 on CD28+ CD45RA+ CD8+ T cells, and HLA DR++ monocyte %leukocyte was negatively correlated with the risk of AK, indicating that an increase in the abundance of these immune cells leads to a decrease in AK risk. HLA DR on monocyte (OR=0.864, 95% CI=0.808-0.924), HLA DR on dendritic cell (OR=0.909, 95% CI=0.867-0.952), CD28 on CD28+ CD45RA+ CD8+ T cell (OR=0.804, 95% CI=0.722-0.896), HLA DR++ monocyte %leukocyte (OR=0.735, 95% CI=0.630-0.857).
		d)	Consider plots to visualize results (e.g. forest plot, scatterplot of associations between genetic variants and outcome versus between genetic variants and exposure)		The results of the forest plots are shown in figure 3, figure 4 and figure 5. The results of the scatter plot and leave-one-out plot are detailed in the Supplementary Materials.
12	**Assessment of assumptions**				
		a)	Report the assessment of the validity of the assumptions	3	To identify SNPs associated with the exposure factor and ensure the reliability and accuracy of conclusions regarding the relationship between

					immune cells and skin cancer risk, the following steps were taken to select the most optimal SNPs. Initially, acknowledging that only a limited number of SNPs in the immune cell group met the genome-wide significance threshold (p<5 × 10-8). Additionally, to ensure the independence of the selected instrumental variables (IVs) and minimize bias resulting from residual linkage disequilibrium of genetic variations, we employed the two-sample MR R package with a distance set at 10,000 kb and a linkage disequilibrium threshold of r^2<0.001. On top of that, to mitigate the potential bias resulting from weak instrumental variables, the F-statistic was utilized to assess the statistical strength of the correlation between each SNP and the exposure. IVs with an F-statistic exceeding 10 were considered strong instruments, while those with an F-statistic less than 10 indicated a weak correlation between SNPs and the exposure. During the analysis, SNPs with palindromic structures were automatically excluded.
		b)	Report any additional statistics (e.g., assessments of heterogeneity across genetic variants, such as I^2, Q statistic or E-value)	3	We employed Cochran's Q method to assess the heterogeneity of the selected IVs. A significant result (p<0.05) would indicate significant heterogeneity in the analysis outcomes. To reduce the influence of horizontal pleiotropy, we utilized the MR-Egger regression test. The results showed p-values greater than 0.05, indicating the absence of heterogeneity and pleiotropy in the SNPs (Table 1 and Table 2).
13	**Sensitivity analyses and additional analyses**				
		a)	Report any sensitivity analyses to assess the robustness of the main results to violations of the assumptions	3-5	In this study, we applied five MR methods to validate the causal associations between genetic variations in immune cells and skin cancer. The methods comprised inverse variance weighted analysis (IVW), weighted median (WM), simple median (SM), weighted median estimator (WME), and MR-Egger regression. In the analysis, the IVW method, based on genotype summary data, served as the primary approach. The IVW method combined Wald estimates for each single nucleotide polymorphism through a meta-analysis,

				resulting in an overall estimate. The weighted regression slope of the effect of the result on the effect of the exposure, with the intercept constrained to zero, represented the overall estimate. This approach provided a comprehensive evaluation of the causal relationship between genetic variations in immune cells and skin cancer (The results are shown in figure 3, figure 4 and figure 5).
	b)	Report results from other sensitivity analyses or additional analyses	5	In the sensitivity analysis, we conducted heterogeneity and pleiotropy analyses for the immune cells included in the study and their corresponding skin cancer diseases. The results showed p-values greater than 0.05, indicating the absence of heterogeneity and pleiotropy in the SNPs (Table 1 and Table 2).
	c)	Report any assessment of direction of causal relationship (e.g., bidirectional MR)	3-5	We discovered that potential causal relationships between different types of immune cells and skin cancer disease. Specifically, one type of immune cell as potentially causal to MM, eight different types of immune cells as potentially causal to BCC, four different types of immune cells as potentially causal to AK. In the reverse MR analysis of immune cells and skin cancer, all MR results were greater than 0.05, indicating that skin cancer had no impact on the included immune cells (The results are detailed in the Supplementary Materials).
	d)	When relevant, report and compare with estimates from non-MR analyses	1	Numerous meta-analyses and clinical studies have shown that subtypes of immune cells are associated with the development of skin cancer, but it is not clear whether this association is causal or biased. Mendelian randomization (MR) analysis reduces the effect of confounding factors and improves the accuracy of the results when compared to traditional studies.
	e)	Consider additional plots to visualize results (e.g., leave-one-out analyses)	5	Leave-one-out method refers to the gradual elimination of each SNP, calculating the meta effect of the remaining SNPs, and observing whether the results change after the elimination of each SNP; if the results change a lot after the elimination of a certain SNP, it means that the existence of a certain SNP has a great influence on the results, and the overall error line does not

				change much after the elimination of each SNP (all error lines are to the right of 0 or all error lines are to the left of 0), indicating that the results are stable(leave-one-out plot are detailed in the Supplementary Materials).
	**DISCUSSION**			
14	**Key results**	Summarize key results with reference to study objectives	5-6	We discovered that potential causal relationships between different types of immune cells and skin cancer disease. Specifically, one type of immune cell as potentially causal to malignant melanoma of skin, eight different types of immune cells as potentially causal to basal cell carcinoma, four different types of immune cells as potentially causal to actinic keratosis, and no different types of immune cells were found to have a potential causal association with squamous cell carcinoma. This may provide new insights for researchers to explore the immunology of skin cancer pathogenesis and help to explore early intervention and therapeutic approaches.
15	**Limitations**	Discuss limitations of the study, taking into account the validity of the IV assumptions, other sources of potential bias, and imprecision. Discuss both direction and magnitude of any potential bias and any efforts to address them	7	The study has certain limitations, first, due to a lack of individual-level information, further stratified analysis within the general population was not feasible. Second, immune cell data and skin cancer data were derived from different studies, introducing some differences in sample size, quality control methods, and racial composition, which might lead to some errors. Third, the majority of participants in the GWAS data used in this study were of European ancestry, potentially impacting the generalizability of the findings to other ethnic groups.
16	**Interpretation**			
	a)	Meaning: Give a cautious overall interpretation of results in the context of their limitations and in comparison with other studies	5	These Mendelian randomization analyses suggest that the potential causal relationships between various immune cells and different skin cancer.
	b)	Mechanism: Discuss underlying biological mechanisms that could drive a potential causal relationship between the investigated exposure and the outcome, and whether the gene-environment equivalence assumption is reasonable. Use causal language carefully, clarifying that IV estimates may provide causal effects only under certain assumptions	5	CD25 is primarily involved in the differentiation and proliferation of regulatory CD4+ T cells. In CD4+CD25+ Tregs cells, CD25 serves as a crucial component of the IL-2 receptor, inducing structural changes in IL-2, thereby promoting the formation of the IL2R α / β / γ and IL-2 tetramer, activating

				JAK/STAT5, PI3K/Akt/mTOR, and mitogen-activated protein kinase (MAPK) signaling pathways, enabling Treg cells to exert immune-regulatory functions.While CD25 is primarily expressed on activated T cells, it is also expressed in some B cell subgroups. On B cells, CD25 acts as an activation marker, indicating that B cells have been stimulated and have entered an activated state. Activated B cells can produce specific antibodies or IL-2. In the TME, they participate in M1 cell polarization and recruit effector T cells, playing an anti-cancer role. The MR analysis in this study showed a negative correlation between CD25-labelled B cells and the risk of MM. However, research on the anti-cancer mechanisms of CD25-marked B cells is currently lacking. Therefore, the conclusions of this study provide a theoretical foundation for subsequent research.
	c)	Clinical relevance: Discuss whether the results have clinical or public policy relevance, and to what extent they inform effect sizes of possible interventions	6	After the first development of tumor immunotherapies, the understanding of tumor resistance and immunosuppression has gradually deepened, and the demand for personalized and precise medicine has gradually increased, and immunotherapy has become a major breakthrough in the field of cancer treatment. However, despite the impressive achievements of immunotherapies such as immune checkpoint inhibitors and CAR-T cell therapy, only a small number of patients are still able to benefit from current immunotherapies, and many patients become resistant to treatment or fail to produce the expected response, which can be partly explained by differences in immune subtypes. Therefore, by comprehensively analyzing the immune subtypes of tumors, gaining a deeper understanding of the interactions between tumors and the immune system, and exploring the molecular mechanisms of the immune response, we can more accurately predict the response of patients to specific immune therapies, and thus guide a more personalized therapeutic strategy.
17	**Generalizability**	Discuss the generalizability of the study results (a) to other populations, (b) across other exposure periods/timings, and (c) across other levels of exposure	7	Although most participants in the GWAS summary data used in our study are of European descent, this may partly affect our estimates, and therefore, the conclusions cannot be extended to other racial groups, limiting the generalizability of our results. Secondly, due to the lack of individual information,

				we could not conduct further stratified analysis of the population. Therefore, further research is needed to validate these findings and extend them to other populations.
	**OTHER INFORMATION**			
18	**Funding**	Describe sources of funding and the role of funders in the present study and, if applicable, sources of funding for the databases and original study or studies on which the present study is based	9	This work was supported by Yunnan Province Expert Workstation of Professor Guo jun (Project No.202105AF150038), 2022the Joint Special Funds for the Department of Science and Technology of Yunnan Province-Kunming Medical University （NO.202201AY070001-155） and Scientific Research Fund Project of Education Department of Yunnan Province (No.2023Y0661).
19	**Data and data sharing**	Provide the data used to perform all analyses or report where and how the data can be accessed, and reference these sources in the article. Provide the statistical code needed to reproduce the results in the article, or report whether the code is publicly accessible and if so, where	8	The data used in the present study are all publicly available at https://gwas.mrcieu.ac.uk/. The original contributions presented in the study are included in the article/Supplementary Material.
20	**Conflicts of Interest**	All authors should declare all potential conflicts of interest	8	The authors declare that the research was conducted in the absence of any commercial or financial relationships that could be construed as a potential conflict of interest.

This checklist is copyrighted by the Equator Network under the Creative Commons Attribution 3.0 Unported (CC BY 3.0) license.

1. Skrivankova VW, Richmond RC, Woolf BAR, Yarmolinsky J, Davies NM, Swanson SA, et al. Strengthening the Reporting of Observational Studies in Epidemiology using Mendelian Randomization (STROBE-MR) Statement. JAMA. 2021;under review.

2. Skrivankova VW, Richmond RC, Woolf BAR, Davies NM, Swanson SA, VanderWeele TJ, et al. Strengthening the Reporting of Observational Studies in Epidemiology using Mendelian Randomisation (STROBE-MR): Explanation and Elaboration. BMJ. 2021;375:n2233.
